# New-onset allergic diseases after SARS-CoV-2 infection: mechanistic hypotheses and emerging strategies for risk stratification

**DOI:** 10.3389/fimmu.2026.1879430

**Published:** 2026-06-19

**Authors:** Encheng Li, Manying Shi, Shixiang Huang

**Affiliations:** 1 The Second Affiliated Hospital of Nanchang University, Nanchang, China; 2 The Second School of Clinical Medicine, Nanchang University, Nanchang, China; 3 Jiangxi Provincial Children’s Hospital, Nanchang, China

**Keywords:** allergic diseases, epithelial alarmins, immune tolerance, mast cell hyperactivation, SARS-CoV-2

## Abstract

Multinational cohort studies consistently associate SARS-CoV-2 infection with elevated incidence of allergic diseases, with hazard ratios of 2.25 for asthma and 1.23 for allergic rhinitis persisting beyond six months post-infection; whether this excess risk reflects *de novo* allergic sensitization or preferential unmasking of pre-existing subclinical atopy remains to be established. Yet mechanisms bridging acute viral illness to delayed allergic phenotypes remain incompletely understood. This review synthesizes recent advances across epithelial biology, immunology, and neuroimmune interactions to propose a unified mechanistic framework organized around three interconnected axes. First, epithelial injury during COVID-19 triggers passive IL-33 release while inducing active TSLP and IL-25 production. These alarmins act through mechanistically distinct pathways to converge on type 2 immune priming, which is established and reinforced by epigenetic memory in group 2 innate lymphoid cells and dendritic cells. Second, regulatory T cell depletion and, hypothetically, hematopoietic stem and progenitor cell epigenetic reprogramming driven by acute interleukin-6 elevation may generate immune cell progeny with persistently altered inflammatory responsiveness, while dendritic cells adopt Th2-polarizing phenotypes that lower the threshold for allergic sensitization; the direct contribution of hematopoietic reprogramming to Th2-skewed allergic outcomes remains to be demonstrated. Third, mast cells undergo direct spike protein-mediated activation via angiotensin-converting enzyme 2 receptors, and alarmin-primed mast cells establish bidirectional crosstalk with sensory neurons that amplifies neuroinflammation and links long COVID symptoms to heightened allergic susceptibility. Together, these axes define a post-infectious vulnerability window during which allergen encounters trigger exaggerated type 2 responses. Risk stratification incorporating disease severity, circulating biomarkers including immunoglobulin E and eosinophil counts, and genetic susceptibility variants may identify individuals requiring targeted surveillance, while mechanistically informed interventions such as low-dose interleukin-2, mast cell stabilizers, and alarmin-targeted biologics warrant prospective evaluation in convalescent cohorts.

## 1 Introduction

Allergic diseases now affect an estimated 20–30% of the global population, representing one of the most prevalent categories of chronic immune-mediated conditions worldwide (1). The scale of this burden is substantial: the Global Burden of Disease Study 2021 documents approximately 260 million individuals living with asthma and 129 million with atopic dermatitis (2). These figures, however, obscure a more complex epidemiological picture. Between 1990 and 2021, atopic dermatitis prevalence rose by 20% (3), while asthma incidence declined from 5,568 to 3,340 cases per 100,000 population over the same period (4). A 30-year Polish cohort adds further nuance: childhood asthma prevalence climbed from 3.4% in 1993 to a peak of 12.6% in 2014 before receding to 10.4% by 2023 (5). The divergence between these trends is not merely a statistical curiosity. It signals that allergic disease trajectories are shaped by shifting environmental, behavioral, and immunological pressures that pre-date the COVID-19 pandemic, a distinction that becomes analytically critical when evaluating any pandemic-related contribution to allergic disease incidence.

Against this background, a convergent body of epidemiological evidence now associates SARS-CoV-2 infection with a measurably elevated subsequent incidence of allergic disease in susceptible individuals, though the mechanistic basis of this temporal relationship and the precise contribution of *de novo* sensitization versus unmasking of pre-existing atopy remain incompletely resolved. Oh et al. examined multinational cohorts from South Korea, Japan, and the United Kingdom, reporting hazard ratios of 2.25 (95% CI 1.80–2.83) for new-onset asthma, 1.23 (95% CI 1.15–1.32) for allergic rhinitis, and 1.15 (95% CI 0.96–1.37) for atopic dermatitis following SARS-CoV-2 infection (6). Risks peaked within three months post-infection yet remained elevated beyond six months. This persistence implies that whatever immune perturbation drives these outcomes is not simply resolved with viral clearance (6). US cohort data independently confirmed elevated risks for asthma (HR 1.656) and allergic rhinitis (HR 1.272), with infection conferring two- to three-fold greater risks than vaccination alone (7). The breadth of immune-mediated sequelae extends further still: Gil et al., in a systematic review and meta-analysis, identified elevated risks for at least 11 distinct immune-mediated conditions following COVID-19, suggesting that the immunological disruption involved is not organ-specific but systemic (8).

These associations, while statistically robust and independently replicated, carry methodological caveats that fundamentally constrain causal inference and that this review’s mechanistic framework must explicitly acknowledge rather than assume away. The absence of baseline allergic status assessment in most studies raises the possibility that subclinical sensitization was rendered clinically manifest by infection, rather than representing genuine *de novo* allergic development (9). Follow-up periods of 3–24 months across the majority of cohort studies are insufficient to determine whether the observed risk elevations reflect transient immune perturbations or durable alterations in immune architecture (10). Crucially, the inter-study variability is itself informative: the gap between Oh et al.’s HR of 2.25 and the US cohort’s HR of 1.656 for asthma likely reflects genuine differences in population genetics, healthcare ascertainment, and prior allergic burden rather than noise. This heterogeneity implies that the risk is not uniformly distributed and that individual-level vulnerability factors exist.

This heterogeneity defines the central scientific problem. Why do some SARS-CoV-2-infected individuals subsequently develop allergic disease while the majority recover uneventfully? What immunological mechanisms account for the temporal dissociation between acute respiratory infection and allergic phenotypes emerging months later at distant mucosal sites, including the skin, nasal mucosa, and gastrointestinal tract? And what sustains this vulnerability beyond the period of active viral replication? Recent advances in COVID-19 immunology point to profound and durable immune reprogramming across multiple cellular compartments: epithelial barrier disruption, regulatory T cell depletion, dendritic cell functional skewing, and mast cell hyperactivation emerge as recurring themes. Yet these observations have largely been studied in isolation, without a framework that explains how they interact temporally to lower the threshold for allergic sensitization.

This review synthesizes current evidence into a mechanistic framework addressing these gaps. While prior work has examined individual aspects of post-COVID-19 immune dysregulation, including Filippatos et al.’s recent synthesis of allergy predisposition mechanisms in children (11), the present review departs from existing literature in two respects. First, it integrates evidence across three mechanistic axes into a temporally structured, unified framework that explicitly maps how these pathways interact to create a post-infectious vulnerability window for allergic sensitization. Second, it extends this mechanistic synthesis to translational endpoints, proposing a biomarker-informed risk stratification framework and identifying specific, hypothesis-driven intervention candidates for prospective evaluation in convalescent cohorts, a dimension absent from existing mechanistic reviews.

The present review examines these three interconnected pathways: 1. epithelial alarmin release driving type 2 immune priming; 2. immune tolerance disruption through regulatory T cell and dendritic cell dysfunction; 3. mast cell-mediated neuroimmune sensitization. These axes form an integrated network maintained through cytokine crosstalk, epigenetic memory, and metabolic reprogramming, collectively creating an ‘Immune Memory Imprint’ that may lower thresholds for allergic sensitization (11). Addressing these knowledge gaps will require coordinated multidisciplinary efforts spanning clinical epidemiology, systems immunology, and translational therapeutics.

## 2 Epithelial-derived alarmins in COVID-19 and allergic disease

Epithelial-derived alarmins, namely interleukin-33 (IL-33), thymic stromal lymphopoietin (TSLP), and interleukin-25 (IL-25), constitute a coordinated molecular triad linking viral-induced epithelial damage to subsequent type 2 immune activation (12). Despite their convergent downstream effects on type 2 immunity, these three alarmins differ substantially in their upstream release mechanisms. IL-33 functions as a classical nuclear alarmin, constitutively expressed and passively released from the nucleus upon necrotic injury or mechanical stress, fitting the canonical DAMP paradigm. TSLP and IL-25, however, are not passively released following cell lysis; rather, they require active, stimulus-driven expression: TSLP is transcriptionally induced *de novo* following viral infection or allergen exposure, while IL-25 is secreted through regulated pathways from tuft cells and airway epithelium. Recognizing these mechanistic distinctions is essential, because they imply different temporal kinetics of alarmin release during COVID-19 — IL-33 surges with the earliest wave of epithelial injury, whereas TSLP and IL-25 elevations may accumulate over a more protracted transcriptional timescale (13). Understanding their integrated roles is essential for elucidating how COVID-19 may predispose to allergic disease development. ACE2-dependent epithelial damage triggers the concurrent release of all three alarmins, which in turn activate ILC2s and dendritic cells to initiate Th2 polarization, forming the core of the epithelial barrier-alarmin axis (14, 15).

### 2.1 IL-33

IL-33 exhibits the most rapid release kinetics among the alarmin triad. Constitutively expressed in epithelial cell nuclei and tethered to chromatin via its chromatin-binding domain, IL-33 is passively released within minutes following necrosis or mechanical stress (16). During acute COVID-19, IL-33 levels correlate significantly with disease severity. Wang et al. demonstrated that elevated IL-33 amplifies inflammatory cascades through NF-κB and p38 MAPK pathways, driving production of IL-1β, IL-6, and TNF-α, and IL-33-deficient mice infected with SARS-CoV-2 showed both reduced viral burden and attenuated lung pathology (17). Scott et al. further established that IL-33 promotes alveolar epithelial dysfunction during viral respiratory infection, identifying ST2-dependent signaling as a discrete pathological node separable from its type 2 immune functions (18). This functional duality frames a central mechanistic question: why does a molecule indispensable for antiviral defense also predispose to allergic disease months after infection resolution?

The answer lies in IL-33’s capacity to imprint lasting changes on type 2 immune cells. Upon binding ST2, IL-33 triggers MyD88-dependent activation of ILC2s, basophils, mast cells, and Th2 lymphocytes (19). ILC2s respond by rapidly producing IL-5 and IL-13, orchestrating eosinophil recruitment, mucus hypersecretion, and IgE class switching (19). The IL-33-ILC2 axis, however, does more than coordinate acute responses. Martinez-Gonzalez et al. demonstrated that IL-33-experienced ILC2s exhibit markedly enhanced responsiveness upon rechallenge, a memory-like phenotype maintained through H3K4me3 histone marks at type 2 cytokine loci (20). This epigenetically reinforced priming provides a plausible mechanistic explanation for delayed allergic onset following COVID-19: the acute IL-33 surge may render ILC2s hyperresponsive to allergen encounters occurring weeks or months after viral clearance (21). Accordingly, targeting interleukin-33 (IL-33) emerges as a promising therapeutic strategy for managing allergic inflammatory disorders. Anti-IL-33 agents like tozorakimab show promise in asthma trials (22). Whether this priming directly causes new-onset allergic disease or preferentially unmasks pre-existing subclinical sensitization remains an open question that longitudinal immune profiling studies are uniquely positioned to address.

### 2.2 TSLP

TSLP, distinguished by two isoforms (inflammatory long-form and homeostatic short-form), requires *de novo* synthesis following viral infection or allergen exposure (23). Elevated TSLP levels correlate with prolonged hospitalization in COVID-19 (24). Genetic evidence, however, complicates a straightforwardly pro-inflammatory interpretation. Ranjbar et al. demonstrated that TSLP SNPs rs2289276 and rs3806933 associate with lower serum TSLP (p<0.05), with notable sex-specific effects: the influence of rs2289276 predominates in males, while rs3806933 effects are more pronounced in females (25). These promoter variants likely modulate transcriptional efficiency, and their association with reduced TSLP suggests that genetically determined variation in alarmin output may partly account for the individual-level heterogeneity in post-COVID-19 allergic susceptibility described in the Introduction.

Identifying who produces more TSLP is, however, only part of the picture. The downstream consequences depend critically on how TSLP is interpreted by dendritic cells (DCs). Hu et al. demonstrated that TSLP enhances SARS-CoV-2 vaccine responses through DC activation, upregulation of MHC-II and CD80/CD86, and promotion of Tfh cell differentiation; DC-specific TSLPR deletion abrogates these effects, confirming that the DC-TSLP signaling axis is functionally indispensable (26). The same mechanism that supports protective immunity under acute conditions may become maladaptive during prolonged COVID-19 recovery, when sustained TSLP could train DCs to preferentially induce IL-4-producing Tfh cells, thereby lowering the threshold for IgE class switching and allergic sensitization (27). TSLP’s tissue-specific expression pattern adds further clinical relevance to this concern: constitutive production is highest in skin keratinocytes, airway epithelium, and nasal mucosa, precisely the sites where allergic disease is most commonly manifest (28, 29). In atopic dermatitis, keratinocyte-derived TSLP drives basophil activation (30); in asthma and chronic rhinosinusitis, airway TSLP levels correlate with disease severity (31, 32). The therapeutic validation of tezepelumab across multiple allergic conditions confirms TSLP as a legitimate intervention target (33), though whether COVID-19-induced TSLP elevation exhibits tissue-specific persistence capable of predicting organ-specific allergic outcomes remains entirely unexplored.

### 2.3 IL-25

IL-25 (IL-17E) remains the least characterized member of the alarmin triad in the context of COVID-19, despite its well-established potency in initiating type 2 immunity. Structurally belonging to the IL-17 cytokine family but functionally divergent, IL-25 activates ILC2s, Th2 cells, eosinophils, and basophils through IL-17RB (34). In allergic diseases, tuft cell-derived IL-25 initiates the ILC2 activation cascades responsible for goblet cell metaplasia, mucus hypersecretion, and eosinophilic inflammation (35). This functional profile, however, diverges sharply from that of other IL-17 family members: whereas IL-25 (IL-17E) promotes type 2 immunity through ILC2 and Th2 activation, IL-17A and IL-17F, the canonical members elevated in MIS-C, drive Th17-mediated inflammation through overlapping but mechanistically distinct downstream pathways (36, 37). The coexistence within a single cytokine family of both Th2-promoting (IL-25) and Th17-promoting (IL-17A/F) members carries particular interpretive weight in pediatric populations, where MIS-C generates a cytokine milieu dominated by IL-17 rather than the Th2-skewed pattern characteristic of adult post-COVID-19 allergic predisposition. This intra-family divergence may partly account for why pediatric COVID-19 immune sequelae span a broader and more heterogeneous phenotypic spectrum than adult disease, with direct implications for how post-infectious allergic risk should be modeled in children versus adults.

Direct evidence for IL-25 involvement in COVID-19 remains limited to indirect mechanistic inference, and the interpretation of available data requires particular caution. Unlike IL-33 and TSLP, for which human cohort data have established correlations with COVID-19 severity, direct measurement of circulating IL-25 in COVID-19 patients has not been reported in adequately powered studies to date. The involvement of IL-25 in post-COVID-19 immune dysregulation therefore rests on indirect mechanistic reasoning rather than clinical observation. Williams et al. demonstrated that IL-25 blockade augments antiviral immunity during respiratory viral infection by reducing excessive type 2 cytokine responses and preserving interferon-mediated host defenses (38). While this finding does not establish that IL-25 is elevated during acute COVID-19, it does suggest that if IL-25 signaling is amplified during infection, it may simultaneously compromise antiviral clearance and bias the immune environment toward subsequent allergic sensitization — a mechanistic scenario consistent with the alarmin framework proposed in this review.

The tissue distribution of IL-25 introduces an organ-specificity dimension absent from the other two alarmins. IL-25 is markedly enriched in intestinal tuft cells and airway epithelium but shows comparatively low dermal expression (39). This compartmentalization suggests that post-COVID-19 allergic sequelae mediated through the IL-25 axis would be preferentially manifest as gastrointestinal hypersensitivity and respiratory allergies rather than atopic dermatitis, a testable prediction that existing cohort datasets could in principle evaluate. A further regulatory layer deserves attention: Feng et al. reported that tuft cells themselves express IL-17RB, the receptor for IL-25, a configuration that may constitute a negative feedback mechanism restraining local IL-25 bioavailability (40). Whether SARS-CoV-2-induced tuft cell depletion in the gastrointestinal and airway epithelium disrupts this autocrine regulatory circuit, thereby amplifying IL-25 signaling during convalescence, remains uninvestigated and represents a tractable mechanistic hypothesis for future study.

### 2.4 Alarmin triad synergy and COVID-19 to allergy transition

The three epithelial-derived alarmins do not simply add their effects together; they function synergistically, each amplifying the others’ downstream consequences. IL-33 initiates mast cell priming (41), TSLP drives DC maturation and Th2 polarization (42), and IL-25 recruits effector cell populations while sustaining chronic type 2 inflammation (43). All three contribute to rapid ILC2 activation, though IL-25 retains the capacity to engage ILC2s independently of the other two (44). The functional significance of this redundancy is not merely theoretical: combinatorial blockade studies achieve substantially greater suppression of type 2 responses than single-pathway inhibition, a finding that would not hold if the alarmins were operating through truly independent, non-overlapping mechanisms (45). Taken together, this architecture suggests that the post-COVID-19 allergic risk may be disproportionately high in individuals who sustain simultaneous elevation of all three alarmins, rather than transient elevation of any one.

**Figure 1:**
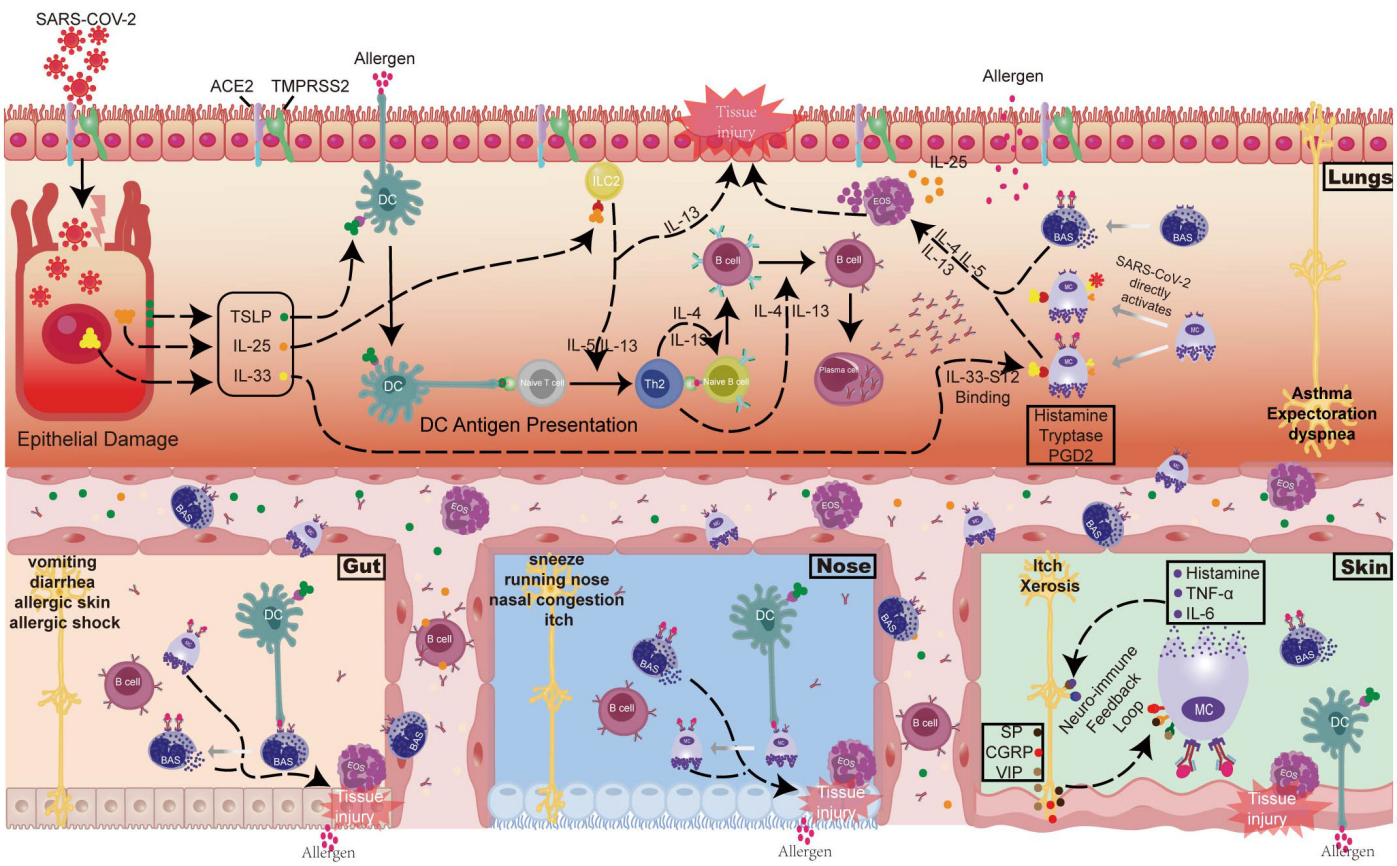
Epithelial alarmin-driven cascade linking SARS-CoV-2 infection to delayed allergic disease development. SARS-CoV-2 enters airway epithelial cells via ACE2 and TMPRSS2 receptors, causing widespread epithelial damage. Injured epithelial cells trigger alarmin release through distinct mechanisms: IL-33 is passively released as a nuclear DAMP upon cell lysis, while TSLP and IL-25 are actively secreted following stimulus-driven transcriptional induction — each with different kinetics but converging on type 2 immune activation. Upper panel (lungs): IL-33 rapidly activates type 2 innate lymphoid cells (ILC2s) and primes mast cells through ST2 signaling, inducing IL-5 and IL-13 production. TSLP instructs dendritic cells (DCs) toward Th2-polarizing phenotypes, upregulating MHC-II and costimulatory molecules. IL-25 recruits eosinophils and sustains type 2 inflammation. These alarmins synergistically activate ILC2s, which release IL-4, IL-5, and IL-13, driving Th2 differentiation. Mast cells undergo degranulation, releasing histamine, tryptase, and prostaglandin D2, establishing neuroinflammatory feedback through histamine-substance P circuits with sensory neurons. Lower panels: Alarmin-primed immune environments create vulnerability windows where subsequent allergen encounters at distant mucosal sites (gut, nose, skin) trigger exaggerated type 2 responses, manifesting as new-onset food allergy, allergic rhinitis, or atopic dermatitis. This integrated pathway explains organ-specific allergic disease development following respiratory viral infection. ACE2, angiotensin-converting enzyme 2; DC, dendritic cell; ILC2, type 2 innate lymphoid cell; Th2, T helper 2 cell; BAS, Basophil; MC, Mast cell; EOS, Eosinophil; SP, substance P; CGRP, calcitonin gene-related peptide; VIP, vasoactive intestinal peptide;PGD2, Prostaglandin D2.

**Figure 1** illustrates the integrated pathophysiological cascade from SARS-CoV-2 infection to allergic disease development. During acute COVID-19, SARS-CoV-2 engages ACE2 and TMPRSS2 to penetrate airway epithelial cells, initiating direct cytopathic injury (46). The resulting epithelial damage triggers coordinated elevation of IL-33, TSLP, and IL-25, which serve initially adaptive functions in antiviral defense and tissue repair (17). Under conditions of prolonged or incompletely resolved epithelial injury extending into convalescence, however, alarmin production is sustained beyond this acute phase. Persistently elevated alarmins stimulate ILC2s to release IL-5 and IL-13 (47), while TSLP-conditioned DCs establish Th2-biased adaptive immunity (26) and IL-25 drives progressive eosinophil accumulation at mucosal surfaces (48). Subsequently, the Downstream cytokines drive multilayered type 2 immune priming (49). The cumulative effect of these converging signals is a remodeled immune landscape characterized by lowered activation thresholds across the ILC2, DC, and mast cell compartments, constituting what may be described a permissive state for allergic sensitization: a measurable shift in immune cell responsiveness in which the barrier between tolerance and overt allergic disease is substantially narrowed. Upon subsequent encounters with environmental allergens, including house dust mites, airborne pollens, and dietary antigens, this pre-primed alarmin-DC-ILC2 network generates exaggerated type 2 responses disproportionate to allergen load (50). IgE class switching accelerates, tissue eosinophilia consolidates, and organ-specific allergic inflammation emerges as new-onset asthma, atopic dermatitis, allergic rhinitis, or food allergy at mucosal sites anatomically remote from the original site of viral injury (51). No published study has comprehensively profiled the temporal dynamics of all three alarmins across sequential COVID-19 stages stratified by subsequent allergic disease development, leaving the predictive weight of individual versus combinatorial alarmin signatures unresolved. Future priorities include prospective cohorts measuring alarmin kinetics, tryptase, and ILC2 phenotypes longitudinally alongside allergic disease screening.

## 3 COVID-19 induced immune tolerance breakdown: direct and indirect evidence

The relationship between COVID-19 and subsequent allergic disease development hinges on understanding how SARS-CoV-2 infection disrupts immune tolerance mechanisms. While direct causal evidence remains limited, converging data from regulatory T cell (Treg) dynamics, dendritic cell (DC) dysfunction, and epigenetic reprogramming of hematopoietic progenitors provide a mechanistic framework linking viral-induced immune perturbations to delayed allergic predisposition.

### 3.1 Treg dynamics dysfunction: persistent quantitative and qualitative deficits

Regulatory T cells (CD4+CD25+Foxp3+) constitute the primary cellular mediators of peripheral tolerance (52). Flow cytometric analysis of 60 hospitalized COVID-19 patients across ICU and non-ICU cohorts revealed significant reductions in Treg frequency, Foxp3 expression, and IL-10 secretion in both groups, indicating that Treg suppression scales with disease severity (53). More clinically consequential is the persistence of these deficits beyond viral clearance. Aquino et al. reported that Treg subsets failed to normalize one year after infection, with CD45RA+CD62L+ naive Tregs remaining elevated while effector Tregs remained depleted (p < 0.01) (54). This sustained dysregulation contrasts with typical post-viral recovery, where Treg homeostasis restores within weeks (55).

### 3.2 Epigenetic reprogramming of hematopoietic stem cells

Cheong et al.’s 2023 Cell study provides the most direct evidence to date for durable epigenetic reprogramming of hematopoietic stem and progenitor cells (HSPCs) in severe COVID-19 survivors, with documented consequences for myeloid innate immune cell function persisting months after viral clearance. Two important limitations must be stated at the outset: this study examined myeloid, not lymphoid, progenitor reprogramming, and it did not assess Th2 polarization, IgE production, or any allergic outcome. Its relevance to post-COVID-19 allergic predisposition therefore rests on indirect mechanistic inference rather than demonstrated pathophysiology (56). A complementary epigenomic and transcriptomic analysis of monocytes from severe COVID-19 patients revealed upregulation of S100A8 alongside concurrent downregulation of tolerance-associated gene expression, suggesting that the epigenetic changes extend beyond inflammatory amplification to active suppression of tolerance programs (57). Brauns et al. further demonstrated that monocytes from acute and convalescent patients display distinct chromatin accessibility profiles that persist weeks after viral clearance, corroborating the interpretation that these are not transient activation states (58).

IL-6, elevated during acute COVID-19, appears to be a proximate driver of this reprogramming: mice treated with anti-IL-6 receptor antibodies exhibited attenuated HSPC epigenetic modifications, providing causal—not merely correlational—evidence for this pathway (56). The implication is significant. HSPCs programmed toward heightened inflammatory responsiveness may persistently generate immune cells with reduced Treg differentiation potential and enhanced pro-inflammatory output for months to years post-infection, a form of trained immunity operating at the stem cell rather than the mature cell level. Whether the epigenetic reprogramming documented in myeloid progenitors extends to lymphoid compartments in a manner that reduces Treg differentiation potential or augments Th2 output remains an open and directly testable question. The present review proposes this as a specific mechanistic hypothesis: that IL-6-driven HSPC reprogramming during severe COVID-19 may alter the lymphoid-myeloid output ratio in ways that reduce Treg reconstitution, a scenario biologically plausible given the shared progenitor pool but as yet undemonstrated in any published study.

### 3.3 Dendritic cell reprogramming: Th2-polarizing phenotypes

DCs determine whether antigen encounters result in tolerance or sensitization, making their functional state a pivotal determinant of post-COVID-19 allergic risk (59). Chang et al. demonstrated that SARS-CoV-2 infection depletes cDC1 and plasmacytoid DCs, with surviving DCs exhibiting downregulated HLA-DR and reduced CD80/CD86 expression (60). Sanchez-Cerrillo et al. found that CD1c+cDC (Th2-promoting) accumulate preferentially in COVID-19 patient lungs while CD141+cDC (Th1-promoting) remain depleted (61). This selective enrichment is not a passive consequence of inflammatory attrition; rather, it reconfigures the antigen-presentation environment so that subsequent allergen encounters occur in a DC landscape already skewed toward type 2 instruction. Direct SARS-CoV-2 infection via DC-SIGN receptors and IL-6–mediated pro-inflammatory DC reprogramming have both been implicated as contributing mechanisms (60, 62), suggesting that the DC phenotypic shift is driven by at least two parallel pathways and is unlikely to resolve rapidly upon viral clearance.

### 3.4 A three-phase model bridging COVID-19 to allergic disease

**Figure 2:**
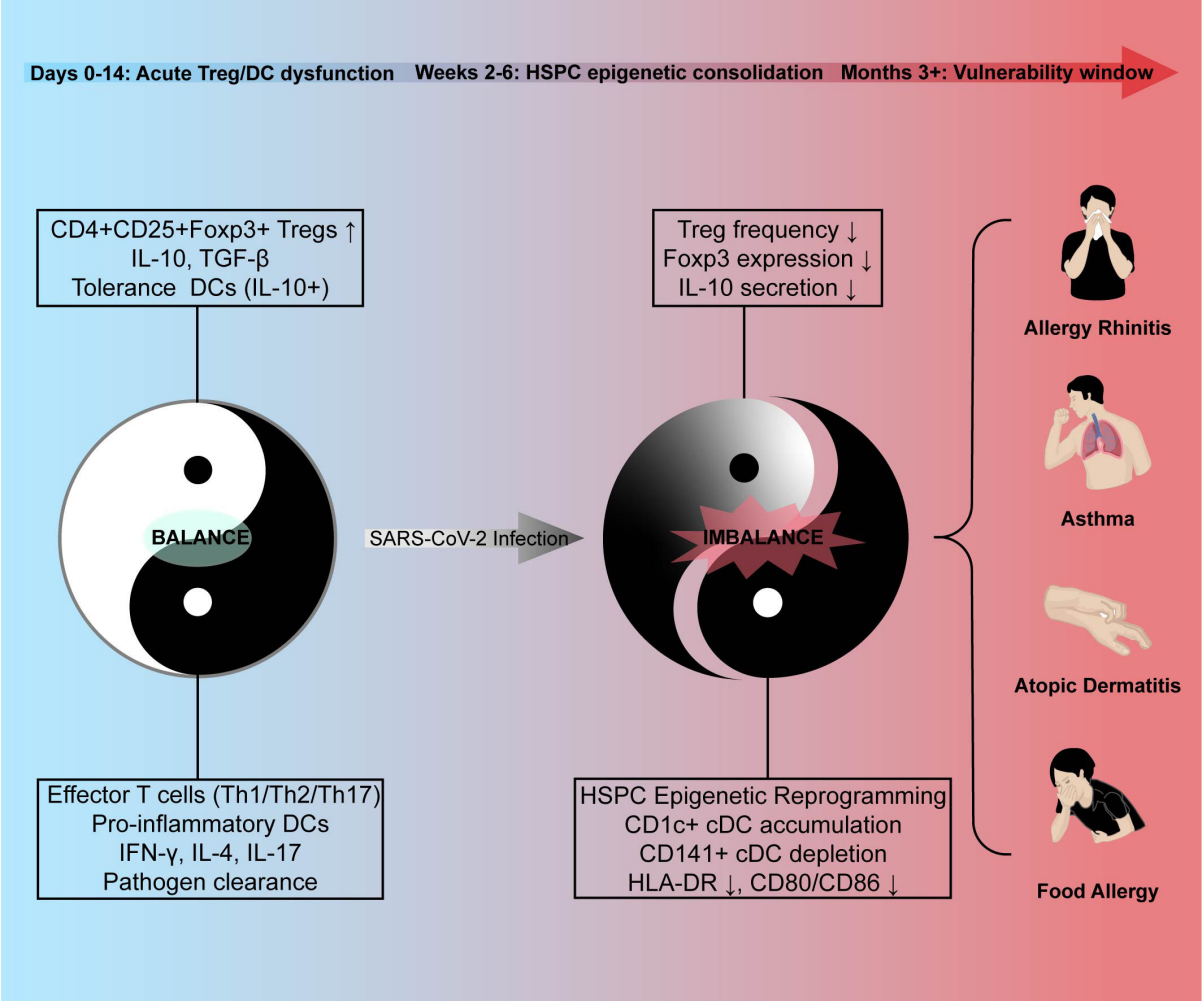
Immune tolerance balance to imbalance (Yin-Yang diagrams). Yin-Yang diagrams depict immune balance shift following SARS-CoV-2 infection (Yin-Yang diagram represents a balance in China). Left: Normal homeostasis maintains equilibrium between tolerance (white, Tregs, IL-10/TGF-βetc.) and effector responses (black, Th1/Th2/Th17 etc.). Right: Post-infection dysregulation features tolerance collapse (shrinking white area) and type 2 dominance (expanding black gradient), driven by Treg depletion and DC Th2-polarization, with HSPC epigenetic reprogramming proposed as a contributing mechanism pending direct lymphoid lineage evidence (CD1c^+^ accumulation, CD141^+^ depletion, HLA-DR↓etc.). Timeline: Acute dysfunction (Days 0-14) progresses through epigenetic consolidation (Weeks 2-6) to a vulnerability window (Months 3+). Outcomes: New-onset asthma, allergic rhinitis, atopic dermatitis, and food allergy emerge during this window. Blue-to-red gradient represents transition from controlled to pathological immunity. Treg, regulatory T cell; DC, dendritic cell; HSPC, hematopoietic stem and progenitor cell.

Integrating these findings suggests a temporal model (**Figure 2**). During Phase 1 (acute infection, days 0–14), SARS-CoV-2–induced epithelial damage triggers simultaneous disruption of Treg frequency and DC function against a cytokine storm milieu of IL-6, IL-1β, and TNF-α. Phase 2 (recovery, weeks 2–6) is defined not by immune normalization but by consolidation of epigenetic HSPC reprogramming: sustained IL-6 has been shown to train HSPCs toward myeloid inflammatory reprogramming (56); whether this reprogramming also impairs lymphoid Treg output remains undemonstrated, but constitutes a testable prediction of the model presented here. Concurrently, DCs recover numerically yet retain Th2-skewing functional properties (61), a finding with more direct evidential support. Tolerance mechanisms remain quantitatively present but qualitatively compromised. In Phase 3 (post-convalescence, from approximately month 3 onward), allergen encounter occurs against this remodeled immune background: deficient Treg suppression fails to restrain allergen-specific IgE production, Th2-biased DCs amplify allergen-specific Th2 differentiation, and new-onset asthma, atopic dermatitis, or allergic rhinitis emerges clinically (**Figure 2**).

Indirectly supporting this model, Oh et al. reported peak post-COVID-19 allergic disease incidence within three months post-infection, with risk persisting throughout at least six months of follow-up (6). Elevated total IgE in COVID-19 convalescents relative to controls provides additional circumstantial support (63).

Despite this framework, direct causal validation is absent. No study has prospectively tracked immune tolerance biomarkers alongside longitudinal allergy screening. Key missing evidence includes: 1. whether greater Treg deficits predict higher allergy incidence; 2. whether HSPC alterations are SARS-CoV-2–specific rather than generic severe illness consequences; 3. whether Treg-enhancing interventions prevent post-COVID-19 allergies. Future priorities include: a. prospective cohorts with serial Treg/DC/ILC2 measurements and allergy screening; b. animal studies testing whether Treg depletion or HSPC reprogramming is necessary for enhanced allergic sensitization; c. interventional trials testing immune tolerance restoration strategies in high-risk COVID-19 convalescents.

The pediatric population warrants specific consideration within this framework. Multisystem Inflammatory Syndrome in Children (MIS-C), a severe post-infectious hyperinflammatory condition occurring 2–6 weeks after SARS-CoV-2 infection, represents the most extreme manifestation of post-COVID-19 immune dysregulation in children and provides a high-magnitude model for studying the mechanisms described above (36). Immunophenotypic analyses reveal Treg depletion, lymphopenia, and marked elevation of IL-6, IL-17, TNF-α, and IFN-γ, a cytokine profile that partially overlaps with, yet remains distinct from, the Th2-dominant pattern observed in adult post-COVID-19 allergic disease (36). Using 13-color flow cytometry on spike-antigen-stimulated PBMCs from unvaccinated children, Filippatos et al. demonstrated significant IL-17 polarization in acute MIS-C: CD4+IL-17+/million CD3+ values were 293.0 (IQR 256.4–870.9) versus 96.7 (IQR 89.2–135.4) in convalescent COVID-19 children (p=0.03), with parallel elevation of CD8+IL-17+ and CD8+IFNγ+ populations (37). Longitudinal profiling by Rybkina et al. further showed that children recovering from MIS-C retained higher frequencies of virus-specific pro-inflammatory memory T cells than those with uncomplicated COVID-19, suggesting that Th17-skewed immune imprinting may persist well into convalescence (64). Whether this IL-17-dominant post-MIS-C immune landscape confers distinct allergic risk trajectories, potentially through Th17/Th2 co-activation or IL-17-amplified epithelial barrier disruption, compared to the adult Th2/Treg-centric model proposed in this review remains an open question that future longitudinal pediatric cohort studies are well positioned to address.

## 4 Mast cell hyperactivation: the intersection of long COVID and allergic disease

Mast cells (MCs), positioned strategically at host-environment interfaces, have emerged as pivotal bridging cells connecting acute COVID-19 to subsequent allergic disease development (65, 66). Recent experimental and histopathological evidence indicates that SARS-CoV-2 infection can trigger substantial MC accumulation and sustained activation, a state that may mechanistically contribute to the transition from acute COVID-19 to delayed allergic manifestations. The term ‘mast cell activation syndrome’ is used here to describe this biological phenomenon of persistent MC hyperactivation rather than as a formal clinical diagnosis, as whether this state meets established diagnostic criteria for MCAS in COVID-19 convalescents has not been systematically evaluated (67).

### 4.1 SARS-CoV-2 direct activation of mast cells

SARS-CoV-2 directly activates MCs through IgE-independent pathways. Wu et al. demonstrated that spike protein’s receptor-binding domain (RBD) triggers MC degranulation within minutes via ACE2 receptors, inducing rapid release of histamine, tryptase, and inflammatory cytokines IL-1β, IL-6, and TNF-α through NLRP3 inflammasome and NF-κB pathway activation (68, 69). Cao et al. confirmed again that spike protein triggers MC degranulation via the cascade in an ACE2-dependent manner (65). Post-mortem examinations of COVID-19 patients reveal substantially increased densities of perivascular MCs with degranulation signatures, corroborating experimental findings (70). This pattern of sustained MC activation generates a proinflammatory microenvironment characterized by elevated histamine, tryptase, and type 2 cytokines, precisely the mediator profile that is known to predispose to allergic sensitization (71).

### 4.2 IL-33/ST2 axis amplification of MC responses

IL-33, markedly elevated during acute COVID-19, synergistically amplifies MC activation through ST2 receptor signaling. Scott et al. (18) demonstrated that lung MCs express high IL1RL1 (ST2 receptor), and IL-33 signaling contributes to alveolar dysfunction in COVID-19 patients. Critically, IL-33 priming lowers MC degranulation thresholds to subsequent stimuli including allergens (72). In COVID-19 contexts, where IL-33 levels correlate with disease severity (17), sustained IL-33 exposure maintains MCs in hyperresponsive states. When individuals encounter environmental allergens during convalescence, these IL-33-primed MCs mount exaggerated degranulation responses.

### 4.3 Neuroimmune crosstalk: MC-sensory neuron bidirectional activation

MCs positioned near sensory nerve fibers engage in bidirectional communication amplifying neuroinflammation and allergic responses (73, 74). MC-released histamine activates H1/H4 receptors on TRPV1^+^ sensory neurons, triggering neuronal activation (75). Tryptase activates PAR2 on 60% of dorsal root ganglion neurons, sensitizing TRPV1 channels and lowering pain/itch thresholds (76). Reciprocally, activated sensory neurons release substance P (SP), calcitonin gene-related peptide (CGRP), and vasoactive intestinal peptide (VIP) that trigger further MC degranulation, establishing positive feedback loops (73).(**Figure 1**) In long COVID patients, persistent neurological symptoms coincide with elevated SP/CGRP in cerebrospinal fluid (77),suggesting sustained neuroimmune activation. This MC-neuron axis may contribute to long COVID symptoms that phenotypically resemble those reported in MCAS, including flushing, pruritus, dysautonomia, and gastrointestinal disturbances (67, 77). The symptom overlap documented by Weinstock et al., in which long COVID patients produced symptom scores statistically indistinguishable from patients undergoing MCAS treatment, is consistent with shared neuroimmune pathophysiology but does not establish MCAS as a diagnosis in the long COVID population (78). This distinction is more than semantic: Lenning et al. conducted a case-control study specifically designed to test for biochemical evidence of MC involvement in long COVID and found no elevation in serum tryptase or urinary MC mediators compared with controls (79), a finding that directly challenges the hypothesis of clinically significant systemic MC activation in this population. The mechanistic framework proposed here is therefore best understood as a biological hypothesis regarding MC priming and neuroimmune amplification, one that accounts for symptom patterns but has not been validated against diagnostic MCAS criteria.

Despite compelling observational data, causal relationships between COVID-19-induced MCAS and subsequent allergic disease require prospective validation. No studies have tracked MC activation biomarkers longitudinally alongside allergy screening. Key missing evidence includes (1): whether observed MC alterations are SARS-CoV-2-specific versus generic post-viral phenomena; (2) whether MC-stabilizing interventions during COVID-19 recovery prevent subsequent allergic disease; (3) whether early MCAS biomarkers identify individuals at highest risk for post-COVID-19 allergies. Future priorities include prospective cohorts with serial tryptase/histamine measurements at 0, 1, 3, 6, and 12 months with concurrent allergy assessments, mechanistic studies using MC-deficient mice in SARS-CoV-2 infection models followed by allergen challenge, and phase II trials testing MC stabilizers for preventing post-COVID-19 allergies in high-risk populations.

## 5 Translational priorities: from mechanistic insight to preventive strategy

The three mechanistic axes described above converge on a shared and immediately practical implication: not all COVID-19 survivors face equivalent risk of subsequent allergic disease. Translating this mechanistic heterogeneity into clinically actionable risk stratification represents the most tractable translational goal at present, even as direct prospective evidence remains limited.

### 5.1 A mechanistically-grounded risk stratification framework

Before engaging with this framework, its evidential status warrants explicit acknowledgment. No validated predictive model exists for post-COVID-19 allergic risk stratification, and none of the biomarker thresholds proposed here have been derived or prospectively tested in COVID-19 convalescent cohorts specifically designed for this purpose. Eosinophil counts, total IgE, serum tryptase, and genetic variants are included on the basis of their mechanistic relevance to the pathways described in this review, not on the basis of demonstrated predictive accuracy in this population. The surveillance intervals proposed in the table are illustrative, anchored to the temporal model of vulnerability described in Section 3.4, and should be treated as hypotheses requiring prospective evaluation rather than as clinical guidelines. The table is intended to operationalize the mechanistic framework into a testable research design, not to direct current clinical practice.

The mechanistic evidence reviewed above can be organized into a preliminary three-tier framework integrating infection severity, convalescent immune biomarkers, and host genetic susceptibility (**Table 1**). This framework is intended as a research hypothesis and study design tool rather than a clinical guideline; its evidential basis and limitations are described in the prefatory note preceding the table. Severe COVID-19 constitutes the strongest upstream risk modifier, reflecting the convergent magnitude of alarmin surges (17, 24), Treg depletion (53), and HSPC epigenetic reprogramming during acute infection (56). Among convalescent biomarkers, sustained peripheral eosinophilia, defined here as an absolute count exceeding 300 cells/μL persisting beyond four weeks post-infection, provides a clinically accessible proxy for ongoing type 2 immune activation (80, 81). Total IgE elevation independently signals enhanced class-switching capacity and warrants longitudinal monitoring (63). Genetic polymorphisms in IL33 (rs3939286) and TSLP (rs2289276, rs3806933) modulate both acute COVID-19 severity and baseline allergic susceptibility (25). These variants may therefore identify individuals whose immunological architecture renders the post-infection vulnerability window particularly permissive, though this inference awaits prospective validation.

**Table 1:** Preliminary risk stratification framework for post-COVID-19 allergic disease.

Risk tier	Clinical profile	Immune biomarker indicators	Proposed surveillance (investigational; not prospectively validated)
Low	Mild or asymptomatic infection; no atopic history; no persistent symptoms	Normal total IgE; eosinophils below 300 cells/μL at 4 weeks	Standard clinical follow-up; symptom-triggered evaluation; no additional surveillance proposed on the basis of current evidence
Moderate	Moderate infection severity, or personal/family atopic history	Eosinophils 300 to 500 cells/μL persisting beyond 4 weeks, or mild IgE elevation; TSLP/IL33 risk genotype	Allergy screening proposed at approximately months 3 and 12 post-infection as mechanistically informed time points; intervals are illustrative pending prospective validation
High	Severe or critical infection requiring hospitalization; MCAS-compatible symptoms including flushing, pruritus, or dysautonomia; multiple concurrent indicators	Eosinophils exceeding 500 cells/μL; markedly elevated total IgE;elevated serum tryptase; TSLP/IL33 risk genotype	Proposed serial IgE and allergen-specific IgE monitoring; skin-prick testing at approximately months 3, 6, and 12 as mechanistically informed time points; specialist referral at clinical discretion. These intervals are illustrative and require prospective validation.

*Several caveats deserve explicit acknowledgment. No validated predictive model yet exists for this specific clinical scenario. The biomarker thresholds proposed here are mechanistically derived rather than prospectively established, and their optimal cutoff values remain undefined. Whether risk-stratified surveillance ultimately modifies allergic disease incidence, or merely enables earlier detection, is an empirical question that prospective cohort studies must address directly.

### 5.2 Mechanistically informed prevention hypotheses

Two mechanistically grounded intervention hypotheses are proposed here, each targeting a distinct node within the three-axis framework. Neither has been evaluated in a COVID-19 convalescent population, and both are presented as candidates for prospective phase II investigation rather than as clinical recommendations. The first concerns low-dose IL-2 (ld-IL-2) therapy, which acts on the Treg deficit that permits unopposed type 2 immune expansion during convalescence. Raeber et al. demonstrated that ld-IL-2 selectively expands CD25^hi Tregs bearing tissue-homing phenotypes capable of migrating to barrier epithelial surfaces, precisely the anatomical interface at which allergic sensitization consolidates (82). A directly testable trial hypothesis follows: if ld-IL-2 initiated during early convalescence, between weeks 2 and 8 post-infection, can restore CD45RA^+^ naive Treg frequency to pre-infection baseline [a measurable endpoint operationalized using Aquino et al.’s published longitudinal cohort data (54)], and if this Treg restoration translates into reduced 12-month allergic disease incidence, that outcome would provide the first prospective evidence linking post-COVID-19 immune tolerance restoration to allergic disease prevention. Whether this causal chain holds is unknown; no interventional data in this population exist.

The second strategy targets the mast cell hyperactivation state with cromolyn sodium and ketotifen, agents that may interrupt the neuroimmune amplification loop hypothesized to consolidate allergic sensitization during the post-COVID-19 vulnerability window. Kazama proposed, in a brief theoretical commentary, that commonly used mast cell-stabilizing drugs may be repositioned for post-COVID symptom relief on the basis of their pharmacological mechanism (83); this represents a hypothesis-generating argument rather than clinical evidence, and no study has tested MC stabilizers specifically for prevention of post-COVID-19 allergic disease. The relevant question here is not whether these agents exert anti-inflammatory effects; that much is pharmacologically established. The critical unknown is whether their administration during the vulnerability window, spanning months 1 through 3 post-infection, reduces 12-month allergic disease incidence in high-risk convalescents. This framing defines a tractable primary outcome for a phase II trial and should guide future study design accordingly.

For both strategies, standardized allergy outcome assessment across 12 to 24 months of follow-up is essential. The temporal dissociation between viral exposure and allergic phenotype emergence means that shorter observation windows will systematically underestimate intervention efficacy, a methodological point that earlier post-viral studies have consistently overlooked.

## 6 Limitations of the present review

This review carries several inherent limitations that constrain the interpretive reach of its conclusions. The three-axis mechanistic framework rests predominantly on indirect and associative evidence; no prospective study has simultaneously tracked alarmin kinetics, Treg dynamics, and mast cell activation biomarkers alongside incident allergic disease in COVID-19 convalescents. The epidemiological foundation, while statistically robust and independently replicated, is subject to ascertainment bias and the absence of baseline atopic phenotyping in most contributing cohorts, leaving the distinction between *de novo* sensitization and unmasking of pre-existing subclinical atopy empirically unresolved. The follow-up periods across available cohort studies, spanning 3 to 24 months, are insufficient to determine whether the observed risk elevations reflect durable immune architectural changes or transient perturbations. The three mechanistic axes are treated here as interacting but analytically separable contributors; their relative weight, potential redundancy, and context-dependence across clinical subgroups, including mild versus severe infection and pediatric versus adult populations, cannot be determined from existing data. Finally, generalization of the adult Th2-centric model to pediatric populations is constrained by the qualitatively distinct cytokine milieu observed in MIS-C, where IL-17A/F dominance rather than Th2 skewing suggests divergent post-infectious immune trajectories that the current framework does not fully accommodate.

## 7 Recommendations for future research

Several research priorities emerge directly from the mechanistic and evidential gaps identified above. Prospective longitudinal cohort studies should simultaneously measure alarmin kinetics, Treg and ILC2 phenotypes, serum tryptase, and allergen-specific IgE at pre-specified time points across the first 12 months post-infection, with allergic disease incidence as the primary outcome and stratification by infection severity. Pediatric cohorts warrant dedicated design, given that MIS-C generates a cytokine profile qualitatively distinct from adult post-COVID immune dysregulation; whether the three-axis framework applies, requires modification, or predicts distinct allergic trajectories in children cannot be determined without population-specific prospective data. Mechanistic animal studies in Treg-depleted or mast cell-deficient mice subjected to standardized SARS-CoV-2 infection followed by allergen challenge would permit causal interrogation of whether each axis is necessary, sufficient, or redundant in producing enhanced allergic sensitization. On the interventional side, phase II trials evaluating low-dose IL-2 initiated during early convalescence and mast cell stabilizers administered during the proposed vulnerability window should incorporate 12-to-24-month allergic disease incidence as a primary endpoint, with serial immune biomarker monitoring as pre-specified secondary outcomes, providing the first prospective test of whether mechanistic plausibility translates into preventive clinical efficacy.

## 8 Conclusion

Converging evidence suggests that SARS-CoV-2 infection may disrupt immune homeostasis through three interconnected mechanisms that, in aggregate, are hypothesized to lower thresholds for allergic sensitization, though this framework, mechanistically plausible as it may be, awaits prospective validation before causal inference can be justified. Epithelial alarmin release establishes type 2 immune priming via ILC2 activation and epigenetic memory. Regulatory T cell depletion sustains quantitative and qualitative deficits in peripheral tolerance for months beyond viral clearance, while hematopoietic stem and progenitor cell epigenetic reprogramming represents a plausible but as yet unvalidated mechanism through which these deficits may be perpetuated. Mast cell hyperactivation, supported by experimental and histopathological evidence but not yet confirmed as a clinically diagnosable entity in COVID-19 convalescents, may amplify neuroimmune circuits in ways that link long COVID symptomatology to heightened allergic predisposition. These pathways converge to create a vulnerability window during which allergen encounters trigger exaggerated responses at distant mucosal sites.

Translation into clinical practice will ultimately require prospective validation of risk stratification tools; the preliminary framework proposed in **Table 1** operationalizes the mechanistic hypotheses of this review into a testable research design, with the expectation that prospective cohort data will refine or revise its biomarker thresholds and surveillance intervals. Prospective cohorts tracking alarmin kinetics, Treg dynamics, and mast cell activation markers alongside longitudinal allergy screening will determine whether mechanistic insights predict individual risk. Prospective interventional trials testing low-dose IL-2, mast cell stabilizers, and alarmin-targeted biologics during convalescence would be the necessary next step to evaluate whether mechanistic plausibility translates into clinical efficacy. Current evidence justifies heightened clinical vigilance in high-risk populations, supports investment in the prospective cohort infrastructure needed to test these hypotheses, but does not support routine preventive treatment outside the context of a formally designed trial.

The elevated allergic disease risk observed following SARS-CoV-2 infection highlights critical gaps in understanding how respiratory viral infections remodel immune tolerance. Closing these gaps through prospective cohort studies, mechanistic animal models, and appropriately designed interventional trials will yield a generalizable framework for anticipating and mitigating allergic sequelae in the context of future respiratory viral pandemics.
